# Association of *TERT* and *DSP* variants with microscopic polyangiitis and myeloperoxidase-ANCA positive vasculitis in a Japanese population: a genetic association study

**DOI:** 10.1186/s13075-020-02347-0

**Published:** 2020-10-16

**Authors:** Aya Kawasaki, Natsumi Namba, Ken-ei Sada, Fumio Hirano, Shigeto Kobayashi, Kenji Nagasaka, Takahiko Sugihara, Nobuyuki Ono, Takashi Fujimoto, Makio Kusaoi, Naoto Tamura, Kunihiro Yamagata, Takayuki Sumida, Hiroshi Hashimoto, Shoichi Ozaki, Hirofumi Makino, Yoshihiro Arimura, Masayoshi Harigai, Naoyuki Tsuchiya

**Affiliations:** 1 Molecular and Genetic Epidemiology Laboratory, Faculty of Medicine, University of Tsukuba, 1-1-1 Tennodai, Tsukuba, 305-8575 Japan; 2 School of Medical Sciences, University of Tsukuba, Tsukuba, Japan; 3 Department of Nephrology, Rheumatology, Endocrinology and Metabolism, Graduate School of Medicine, Dentistry and Pharmaceutical Sciences, Okayama University, Okayama, Japan; 4 Department of Clinical Epidemiology, Kochi Medical School, Kochi University, Nankoku, Japan; 5 Department of Rheumatology, Graduate School of Medical and Dental Sciences, Tokyo Medical and Dental University, Tokyo, Japan; 6 Department of Lifetime Clinical Immunology, Graduate School of Medical and Dental Sciences, Tokyo Medical and Dental University, Tokyo, Japan; 7 Department of Internal Medicine, Juntendo University Koshigaya Hospital, Koshigaya, Japan; 8 Department of Rheumatology, Ome Municipal General Hospital, Ome, Japan; 9 Department of Rheumatology, Saga University, Saga, Japan; 10 The Center for Rheumatic Diseases, Nara Medical University, Kashihara, Japan; 11 Department of Internal Medicine and Rheumatology, School of Medicine, Juntendo University, Tokyo, Japan; 12 Department of Nephrology, Faculty of Medicine, University of Tsukuba, Tsukuba, Japan; 13 Department of Internal Medicine, Faculty of Medicine, University of Tsukuba, Tsukuba, Japan; 14 School of Medicine, Juntendo University, Tokyo, Japan; 15 Department of Internal Medicine, St. Marianna University School of Medicine, Kawasaki, Japan; 16 Okayama University, Okayama, Japan; 17 Department of Nephrology and Rheumatology, Kyorin University School of Medicine, Mitaka, Japan; 18 Department of Internal Medicine, Kichijoji Asahi Hospital, Musashino, Japan; 19 Department of Rheumatology, School of Medicine, Tokyo Women’s Medical University, Tokyo, Japan

**Keywords:** Myeloperoxidase-ANCA, Vasculitis, Microscopic polyangiitis, Susceptibility, Single nucleotide variant, Polymorphism

## Abstract

**Background:**

Interstitial lung disease (ILD) is a severe complication with poor prognosis in anti-neutrophil cytoplasmic antibody (ANCA)-associated vasculitis (AAV). Prevalence of AAV-associated ILD (AAV-ILD) in Japan is considerably higher than that in Europe. Recently, we reported that a *MUC5B* variant rs35705950, the strongest susceptibility variant to idiopathic pulmonary fibrosis (IPF), was strikingly increased in AAV-ILD patients but not in AAV patients without ILD; however, due to the low allele frequency in the Japanese population, the *MUC5B* variant alone cannot account for the high prevalence of AAV-ILD in Japan. In this study, we examined whether other IPF susceptibility alleles in *TERT* and *DSP* genes are associated with susceptibility to AAV subsets and AAV-ILD.

**Methods:**

Five hundred and forty-four Japanese patients with AAV and 5558 controls were analyzed. Among the AAV patients, 432 were positive for myeloperoxidase (MPO)-ANCA (MPO-AAV). A total of 176 MPO-AAV patients were positive and 216 were negative for ILD based on CT or high-resolution CT. Genotypes of *TERT* and *DSP* variants were determined by TaqMan SNP Genotyping Assay, and their association was tested by chi-square test.

**Results:**

When the frequencies of the IPF risk alleles *TERT* rs2736100A and *DSP* rs2076295G were compared between AAV subsets and healthy controls, both alleles were significantly increased in microscopic polyangiitis (MPA) (*TERT P* = 2.3 × 10^−4^, *P*_c_ = 0.0023, odds ratio [OR] 1.38; *DSP P* = 6.9 × 10^−4^, *P*_c_ = 0.0069, OR 1.32) and MPO-AAV (*TERT P* = 1.5 × 10^−4^, *P*_c_ = 0.0015, OR 1.33; *DSP P* = 0.0011, *P*_c_ = 0.011, OR 1.26). On the other hand, no significant association was detected when the allele frequencies were compared between MPO-AAV patients with and without ILD.

**Conclusions:**

Unexpectedly, *TERT* and *DSP* IPF risk alleles were found to be associated with MPA and MPO-AAV, regardless of the presence of ILD. These findings suggest that *TERT* and *DSP* may be novel susceptibility genes to MPA/MPO-AAV and also that some susceptibility genes may be shared between IPF and MPA/MPO-AAV.

## Background

Anti-neutrophil cytoplasmic antibody (ANCA)-associated vasculitis (AAV) is a systemic autoimmune disease characterized by ANCA production and inflammation in small vessels [1]. There are substantial differences in the epidemiology of AAV between European and Asian populations. Microscopic polyangiitis (MPA) and myeloperoxidase (MPO)-ANCA positive AAV (MPO-AAV) are predominant in East Asian populations, while granulomatosis with polyangiitis (GPA) and proteinase 3 (PR3)-ANCA positive AAV (PR3-AAV) are common in the populations of European ancestry [2]. Another striking difference is that the prevalence of AAV-associated interstitial lung disease (AAV-ILD), a complication associated with poor prognosis, is considerably higher in Japanese than in European populations [3]. Among the AAV subsets, ILD was predominantly observed in MPA and MPO-AAV as compared with GPA and PR3-AAV [3]; however, significantly higher complication rate of ILD in the Japanese than in the European population is also observed when only MPA patients were compared [4], suggesting that genetic factors may contribute to the susceptibility to AAV-ILD.

Genome-wide association studies (GWAS) on AAV have been reported in European populations, and associations of *HLA-DP*, *SERPINA1*, *PRTN3*, and *PTPN22* with GPA and PR3-AAV; *HLA-DQ* and *PTPN22* with MPA and MPO-AAV; and *HLA-DQ*, *BCL2L11*, and *TSLP* with eosinophilic granulomatosis with polyangiitis (EGPA) were identified [5–8]. In a Japanese population, we reported that HLA-DRB1*09:01-DQB1*03:03 haplotype and DRB1*13:02 were associated with risk and protection for MPA/MPO-AAV, respectively [9, 10]. However, genetic factors of AAV have not been fully determined.

Little is known on the genetic factors associated with the occurrence of ILD among the patients with autoimmune rheumatic diseases. Recently, we reported that a single nucleotide variant (SNV) rs35705950 in the upstream region of *MUC5B* gene, the strongest susceptibility variant to idiopathic pulmonary fibrosis (IPF) [11–13], was associated with ILD in the patients with rheumatoid arthritis (RA) in a multinational collaborative study [14]. Subsequently, we also reported association of rs35705950 with AAV-ILD [15]. Based on the histological and radiographic patterns of idiopathic interstitial pneumonia (IIP), ILD in AAV and RA is most frequently classified into usual interstitial pneumonia (UIP), typically observed in IPF [3]. These findings suggest a possibility that there may be shared pathological processes between IPF and AAV-ILD.

Although the association between *MUC5B* rs35705950 and AAV-ILD is striking (odds ratio [OR] 11.6 when compared with AAV patients without ILD) [15], this allele alone cannot account for the high complication rate of ILD in Japanese AAV, because the population frequency of the risk allele is substantially lower as compared with European populations [14, 15]. Thus, other genetic factors are likely to play a role in the occurrence of ILD among AAV patients in Japan.

In addition to *MUC5B*, *TERT* and *DSP* have been reported to be associated with IPF and IIPs in GWAS [11–13, 16]. *TERT* gene encodes telomerase reverse transcriptase, which is the catalytic subunit of telomerase, and contributes to maintenance of telomere length [17]. Desmoplakin, encoded by *DSP*, is one of desmosomal components and has a role in cell-cell adhesion and tissue integrity [18]. Although the mechanisms by which these susceptibility genes contribute to development of IPF remain unclear, *TERT* and *DSP* could be considered candidate genes which might be associated with ILD among the patients with AAV, in a similar manner to *MUC5B*.

This study was carried out to examine whether the IPF risk SNVs in *TERT* and *DSP* genes are associated with AAV subsets and presence of ILD among AAV patients in a Japanese population. Unexpectedly, *TERT* and *DSP* SNVs turned out to be significantly associated with susceptibility to MPA and MPO-AAV regardless of the presence of ILD.

## Methods

### Patients and controls

Five hundred and forty-four patients with AAV and 785 healthy controls (HC) were analyzed in this study. All patients and controls are unrelated Japanese. The patients were recruited at the institutes participating in Japan Research Committee of the Ministry of Health, Labour, and Welfare for Intractable Vasculitis (JPVAS) and Research Committee of Progressive Renal Disease, both organized by the Ministry of Health, Labour, and Welfare of Japan, and research groups organized by Tokyo Medical and Dental University and University of Tsukuba. The criteria for enrollment were (1) the diagnosis of AAV by site investigators, and (2) fulfilling the entry and exclusion criteria (no other diagnosis to account for symptom/signs, including malignancy, infection, drugs, and secondary vasculitis) for primary systemic vasculitis as proposed by the European Medicines Agency (EMA) algorithm [19]. The patients include those who participated in the two cohort studies previously reported by JPVAS [20, 21]. The AAV patients were classified into 315 MPA, 119 GPA, 73 EGPA, and 37 unclassifiable according to the EMA algorithm [19]. A total of 432 patients were tested positive for MPO-ANCA and 67 for PR3-ANCA by enzyme-linked immunosorbent assay (Table 1). Among the MPO-AAV patients, 176 were positive (hereafter referred to as MPO-AAV-ILD) and 216 were negative for ILD (MPO-AAV-noILD). Diagnosis of ILD was made by site investigators based on computed tomography (CT) or high-resolution CT (HRCT).

**Table 1:** Characteristics in AAV patients and controls

Characteristics	Groups	Number	Female/male
AAV			
EMA classification	MPA	315	192/123
	GPA	119	67/52
	EGPA	73	46/27
	Unclassifiable	37	24/13
ANCA specificity	MPO-AAV	432	268/164
	PR3-AAV	67	33/34
Presence/absence of ILD in MPO-AAV^a^	MPO-AAV-ILD	176	101/75
	MPO-AAV-noILD	216	139/77
Controls	HC^b^	785	478/307
	4.7KJPN^c^	4773	2641/2130

*AAV* ANCA-associated vasculitis, *EMA* European Medicines Agency, *MPA* microscopic polyangiitis, *GPA* granulomatosis with polyangiitis, *EGPA* eosinophilic granulomatosis with polyangiitis, *MPO-AAV* myeloperoxidase (MPO)-ANCA positive AAV, *PR3-AAV* proteinase 3 (PR3)-ANCA positive AAV, *ILD* interstitial lung disease, *MPO-AAV-ILD* MPO-AAV with ILD, *MPO-AAV-noILD* MPO-AAV without ILD

^a^Information of ILD was not available for 40 patients with MPO-AAV

^b^Healthy controls recruited by our research group

^c^Allele frequency data in 4.7KJPN was obtained from the Japanese Multi Omics Reference Panel (jMorp) [22, 23]

Among the 785 HC, 266 samples were purchased from the Health Science Research Resources Bank (Osaka, Japan). In addition, allele frequency data of the *TERT* and *DSP* variants in 4773 individuals (4.7KJPN) was obtained from the Japanese Multi Omics Reference Panel (jMorp) [22, 23]. Together, data in 5558 individuals was used as the control data.

### Ethics statement

This study was reviewed and approved by the Ethics Committees of University of Tsukuba, Tokyo Women’s Medical University, Tokyo Medical and Dental University, and all other institutes participating in this study, and was conducted in accordance with the principles of the Declaration of Helsinki. Informed consent was obtained from all subjects.

### Genotyping

Genotypes of *TERT* rs2736100 (Assay ID: C___1844009_10) and *DSP* rs2076295 (Assay ID: C__16167921_10) were determined by TaqMan SNP Genotyping Assay using 7300 Real-Time PCR System (Thermo Fisher Scientific, Waltham, MA, USA).

### Statistical analysis

Association was tested by chi-square test using two-by-two contingency table. *P* values were corrected for multiple comparison (10 comparisons) by Bonferroni correction. *P* values, corrected *P* values (*P*_c_), odds ratios (ORs), and 95% confidence intervals (CIs) were calculated using R software (version 3.5.2). Power calculation was conducted using PS: Power and Sample Size Calculation version 3.1.6 (http://biostat.mc.vanderbilt.edu/wiki/Main/PowerSampleSize). To calculate the power, the significance level was set at 0.05. The power to detect association with OR = 1.2, 1.4, 1.6, and 1.8 is shown in Additional file 1: Supplementary Table S1.

## Results

### Association of *TERT* and *DSP* IPF risk SNVs with MPA and MPO-AAV

Genotypes for *TERT* rs2736100 and *DSP* rs2076295 were determined in 544 patients with AAV and 785 HC. No deviation from the Hardy-Weinberg equilibrium was observed (rs2736100, AAV: *P* = 0.25, HC: *P* = 0.89; rs2076295, AAV: *P* = 0.27, HC: *P* = 0.91).

Initially, we examined whether *TERT* and *DSP* are associated with susceptibility to AAV subsets. AAV patients were classified according to the EMA algorithm (MPA, GPA, and EGPA) [19], or ANCA specificity (MPO-AAV and PR3-AAV). *TERT* rs2736100A was significantly increased in MPA (*P* = 2.3 × 10^−4^, *P*_c_ = 0.0023, OR 1.38, 95% CI 1.16–1.64) and MPO-AAV (*P* = 1.5 × 10^−4^, *P*_c_ = 0.0015, OR 1.33, 95% CI 1.15–1.54) when compared with controls (Table 2). Similarly, *DSP* rs2076295G was significantly increased in MPA (*P* = 6.9 × 10^−4^, *P*_c_ = 0.0069, OR 1.32, 95% CI 1.12–1.55) and MPO-AAV (*P* = 0.0011, *P*_c_ = 0.011, OR 1.26, 95% CI 1.10–1.45) (Table 2). A tendency towards association of *TERT* rs2736100A with EGPA and PR3-AAV, and that of *DSP* rs2076295G with PR3-AAV was observed, although the association did not reach statistical significance after Bonferroni correction for multiple testing.

**Table 2:** Association of *TERT* and *DSP* with MPA and MPO-AAV

	*TERT* rs2736100A				*DSP* rs2076295G			
	*n* (AF)	*P*	*P*_c_	OR (95% CI)	*n* (AF)	*P*	*P*_c_	OR (95% CI)
AAV								
MPA	427 (0.678)	**2.3 × 10**^**−4**^	**0.0023**	**1.38 (1.16–1.64)**	351 (0.557)	**6.9 × 10**^**−4**^	**0.0069**	**1.32 (1.12–1.55)**
GPA	142 (0.597)	0.82	1	0.97 (0.75–1.26)	130 (0.546)	0.074	0.74	1.26 (0.98–1.64)
EGPA	101 (0.692)	0.031	0.31	1.47 (1.03–2.09)	77 (0.527)	0.34	1	1.17 (0.85–1.63)
MPO-AAV	577 (0.669)	**1.5 × 10**^**−4**^	**0.0015**	**1.33 (1.15–1.54)**	471 (0.545)	**0.0011**	**0.011**	**1.26 (1.10–1.45)**
PR3-AAV	93 (0.694)	0.034	0.34	1.49 (1.03–2.15)	78 (0.582)	0.03	0.3	1.46 (1.04–2.07)
Controls								
HC^a^	965 (0.615)				791 (0.505)			
4.7KJPN^b^	5747 (0.602)				4628 (0.485)			
All controls	6712 (0.604)			Referent	5419 (0.488)			Referent

*P* values were calculated by chi-square test in comparison between each AAV subset and all controls. Correction for multiple testing was done by Bonferroni correction. Corrected *P* values (*P*_c_) were calculated by multiplying uncorrected *P* values by 10. Significant association after correction for multiple testing (*P*_c_ < 0.05) is shown in bold

*AAV* ANCA-associated vasculitis, *n* allele count, *AF* allele frequency, *MPA* microscopic polyangiitis, *GPA* granulomatosis with polyangiitis, *EGPA* eosinophilic granulomatosis with polyangiitis, *MPO-AAV* myeloperoxidase (MPO)-ANCA positive vasculitis, *PR3-AAV* proteinase 3 (PR3)-ANCA positive vasculitis, *HC* healthy controls, *P*_*c*_ corrected *P* values, *OR* odds ratio, *95% CI* 95% confidence interval

^a^Healthy controls recruited by our research group

^b^Allele data in 4.7KJPN was obtained from the Japanese Multi Omics Reference Panel (jMorp) [22, 23]

To exclude the possible effect caused by the difference in female-to-male ratio between AAV and controls, female and male subjects were separately tested for association. As shown in Table 3, the same trend towards association of *TERT* rs2736100A was observed both in the female and male subjects. On the other hand, the association of *DSP* rs2076295G was detected in male (MPA: *P* = 2.3 × 10^−5^, OR 1.76, 95% CI 1.35–2.29; MPO-AAV: *P* = 3.5 × 10^−6^, OR 1.71, 95% CI 1.36–2.15), but not in female individuals.

**Table 3:** Association of *TERT* and *DPS* with AAV in female and male individuals

	*TERT* rs2736100A			*DSP* rs2076295G		
	*n* (AF)	*P*	OR (95% CI)	*n* (AF)	*P*	OR (95% CI)
Female						
MPA	260 (0.677)	0.0014	1.43 (1.15–1.78)	198 (0.516)	0.35	1.10 (0.90–1.36)
MPO-AAV	358 (0.670)	6.0 × 10^−4^	1.39 (1.15–1.67)	269 (0.502)	0.62	1.05 (0.88–1.25)
Controls^a^	3708 (0.595)		Referent	3062 (0.491)		Referent
Male						
MPA	167 (0.679)	0.049	1.32 (1.00–1.73)	153 (0.622)	2.3 × 10^−5^	1.76 (1.35–2.29)
MPO-AAV	219 (0.668)	0.064	1.25 (0.99–1.58)	202 (0.616)	3.5 × 10^−6^	1.71 (1.36–2.15)
Controls^a^	3003 (0.616)		Referent	2355 (0.484)		Referent

*P* values were calculated by chi-square test in comparison between each AAV subset and controls

*AAV* ANCA-associated vasculitis, *MPA* microscopic polyangiitis, *MPO-AAV* myeloperoxidase (MPO)-ANCA positive vasculitis, *n* allele count, *AF* allele frequency, *OR* odds ratio, *95% CI* 95% confidence interval

^a^Data in controls include allele data in healthy controls and 4.7KJPN obtained from the Japanese Multi Omics Reference Panel (jMorp) [22, 23]

### Lack of association of *TERT* and *DSP* IPF risk SNVs with presence of ILD in MPO-AAV

We next investigated whether the *TERT* rs2736100A and *DSP* rs2076295G contribute to the development of ILD in AAV. Because the prevalence of ILD is higher in MPO-AAV compared with PR3-AAV [3], and the *TERT* and *DSP* SNVs were associated with susceptibility to MPO-AAV as described above, only MPO-AAV patients were examined for the association of these SNVs with the presence of ILD in a case-case study comparing MPO-AAV patients with ILD (MPO-AAV-ILD) and those without ILD (MPO-AAV-noILD) (Table 4). No significant association was detected in *TERT* rs2736100 (*P* = 0.37, OR 0.87, 95% CI 0.65–1.18) nor in *DSP* rs2076295 (*P* = 0.52, OR 1.10, 95% CI 0.83–1.46).

**Table 4:** Association study of *TERT* and *DSP* in MPO-AAV with ILD

	*TERT* rs2736100A			*DSP* rs2076295G		
	*n* (AF)	*P*	OR (95% CI)	*n* (AF)	*P*	OR (95% CI)
MPO-AAV-ILD	230 (0.653)	0.37	0.87 (0.65–1.18)	198 (0.563)	0.52	1.10 (0.83–1.46)
MPO-AAV-noILD	294 (0.684)		Referent	233 (0.539)		Referent

*P* values were calculated by chi-square test in comparison between MPO-AAV-ILD and MPO-AAV-noILD. *MPO-AAV* myeloperoxidase-ANCA positive vasculitis, *ILD* interstitial lung disease, *MPO-AAV-ILD* MPO-AAV with ILD, *MPO-AAV-noILD* MPO-AAV without ILD, *n* allele count, *AF* allele frequency, *OR* odds ratio, *95% CI* 95% confidence interval

## Discussion

Here, we report that *TERT* rs2736100A and *DSP* rs2076295G, both of which are the risk alleles for IPF [12, 13, 16], are associated with susceptibility to MPA and MPO-AAV in a Japanese population. Unexpectedly, association of these alleles with occurrence of ILD among the patients with MPO-AAV was not detected. These are new findings which have not been reported in the populations of European nor of Asian ancestry.

It has been recognized that some patients with IPF are positive for MPO-ANCA and develop MPA [24], and hypothesized that IPF and AAV may share some pathogenic mechanisms. Our current finding that the IPF risk alleles of *TERT* and *DSP* are associated with MPA and MPO-AAV is in line with such hypothesis. This is also supported by the reports that silica contributes to the pathogenesis of both IPF and AAV [25, 26].

On the other hand, significant association of *TERT* and *DSP* IPF risk SNVs was not detected when MPO-AAV patients with and without ILD were compared. This finding was unexpected, but similar finding has been reported in RA, where association of these genes was not observed in RA-ILD when compared with RA patients without ILD [14]. This is in contrast to *MUC5B*, which shows association in the patients with AAV and RA only when they are complicated by ILD [14, 15]. These results might suggest a possibility that some IPF associated genes (e.g., *MUC5B*) may play a role in the process of lung disease, and others (e.g., *TERT* and *DSP*) may be involved in the shared molecular background between AAV/RA and IPF. Further studies are required to validate this hypothesis.

The association of *TERT* rs2736100 was reported in GWAS on IPF in a Japanese population [16], and also in GWAS on IIPs, among which IPF was the most common subset, in European populations [12]. *TERT* rs2736100 is located in intron 2 of the *TERT* gene. Wei et al. reported that the *TERT* risk allele, rs2736100A, showed lower enhancer activity compared with rs2736100C, using a luciferase assay in primary lung epithelial cells [27]. They also found that rs2736100A showed decreased expression of *TERT* mRNA [27], suggesting a functional significance of this SNV. Moreover, rs2736100A was associated with shorter leukocyte telomere length [28], previously shown to contribute to risk of IPF [29]. With respect to AAV, a proportion of T cells was reported to show short telomeres in GPA patients [30], although telomere length abnormality has not been reported in MPA or MPO-AAV. Because *TERT* has an anti-apoptotic effect [31], decreased expression of *TERT* associated with the risk allele may result in an enhancement of apoptosis. Indeed, in AAV patients, an enhanced rate of apoptosis was observed in neutrophils [32]. Apoptotic neutrophils may be opsonized with anti-MPO and anti-PR3 antibodies which recognize MPO and PR3 on the surface, engulfed by macrophages, and lead to secretion of pro-inflammatory cytokines such as interleukin-1 and interleukin-8. These processes may induce inflammation in vessel and tissue injury [33, 34].

Interestingly, association of *TERT* rs7726159A has been shown to be associated with systemic lupus erythematosus (SLE) in East Asian populations [35]. The risk SNV for SLE is in moderate linkage disequilibrium (*r*^2^ = 0.773) with that for MPO-AAV and IPF, although the risk allele is the opposite. Nevertheless, this finding may suggest that *TERT* variants may be functional and associated with multiple autoimmune conditions either in a predispositional or in a protective manner, regardless of the presence of ILD.

The association of *DSP* with IIPs and IPF has been identified by GWAS in the European populations [12, 13]. *DSP* rs2076295 is located in intron 5, and the risk allele was reported to be associated with decreased expression of *DSP* in the lung [12]. Expression quantitative trait locus (eQTL) analysis using the GTEx Portal database [36] shows that rs2076295G is the most strongly associated variant with *DSP* expression in the lung (*P* = 3.7 × 10^−75^, normalized effect size = − 0.73, Additional file 2: Figure S1).

DSP was reported to modulate Wnt/beta-catenin signaling, which is involved in cell proliferation, differentiation, immune responses, and carcinogenesis [37, 38]. In *Dsp*-deficient atrial myocyte cell lines and HEK293T cells transfected with *DSP* frameshift variant, the Wnt/beta-catenin signaling was suppressed [39, 40]. Although contribution of the Wnt signaling to the pathogenesis of AAV is unclear, Wnt signaling has been reported to play a role in autoimmune diseases such as SLE and RA [41]. In this study, association of *DSP* was observed in male but not in female AAV. Although the reason of such difference remains unclear, sex hormone or genes located in sex chromosomes might affect the association of *DSP*.

Our study has several limitations. Due to rarity of AAV, a replication study was not conducted. The annual incidence/million in Japan has been reported to be 22.6 and 18.2 for AAV and MPA, respectively [2]. As shown in Additional file 1: Supplementary Table S1, when OR is less than 1.4, we cannot detect the association with power ≥ 0.8 in the case-case analysis comparing AAV patients with and without ILD. Therefore, the results in this study should be confirmed in larger sample size in the future. In addition, not all AAV patients with ILD were diagnosed by HRCT, and lung biopsy was not performed in most of the patients. Thus, we cannot exclude the possibility that ILD with a specific histological pattern such as UIP might show association with *TERT* or *DSP* IPF risk SNVs.

## cc

*TERT* and *DSP* IPF risk SNVs were found to be associated with susceptibility to MPA and MPO-AAV for the first time. On the other hand, significant association with complication of ILD in AAV was not detected. Our findings suggested that some susceptibility genes are shared between IPF and AAV, regardless of the presence of ILD.

## Supplementary information

**Additional file 1: Supplementary Table S1.** Power to detect associations under the sample size in this study.

**Additional file 2: Supplementary Figure S1.** *DSP* rs2076295G is associated with lower expression of DSP mRNA in lung.

## Data Availability

The data that support the findings of this study are included in this published article and its Supplementary Information. Other data are available from the corresponding authors [NT and AK] on reasonable request. However, the genotype data and clinical information of each individual participant are not available, based on the Act on the Protection of Personal Information enforced in Japan and the conditions on which the informed consent was given.

## Abbreviations

AAV: Anti-neutrophil cytoplasmic antibody-associated vasculitis
ANCA: Anti-neutrophil cytoplasmic antibody
CI: Confidence interval
CT: Computed tomography
EGPA: Eosinophilic granulomatosis with polyangiitis
EMA: European Medicines Agency
eQTL: Expression quantitative trait locus
GPA: Granulomatosis with polyangiitis
GWAS: Genome-wide association studies
HC: Healthy controls
HRCT: High-resolution computed tomography
IIP: Idiopathic interstitial pneumonia
ILD: Interstitial lung disease
IPF: Idiopathic pulmonary fibrosis
MPA: Microscopic polyangiitis
MPO: Myeloperoxidase
OR: Odds ratio
PR3: Proteinase 3
RA: Rheumatoid arthritis
SLE: Systemic lupus erythematosus
SNV: Single nucleotide variant
UIP: Usual interstitial pneumonia
