# Supplementary material for: Association of TERT and DSP variants with microscopic polyangiitis and myeloperoxidase-ANCA positive vasculitis in a Japanese population: a genetic association study
Source: Arthritis Res Ther. 2020 Oct 16;22:246. doi: 10.1186/s13075-020-02347-0 (PMC7574242; doi:10.1186/s13075-020-02347-0)
Supplement: Supplementary file 2 — Additional file 2: Supplementary Figure S1. DSP rs2076295G is associated with lower expression of DSP mRNA in lung. [file 13075_2020_2347_MOESM2_ESM.docx]

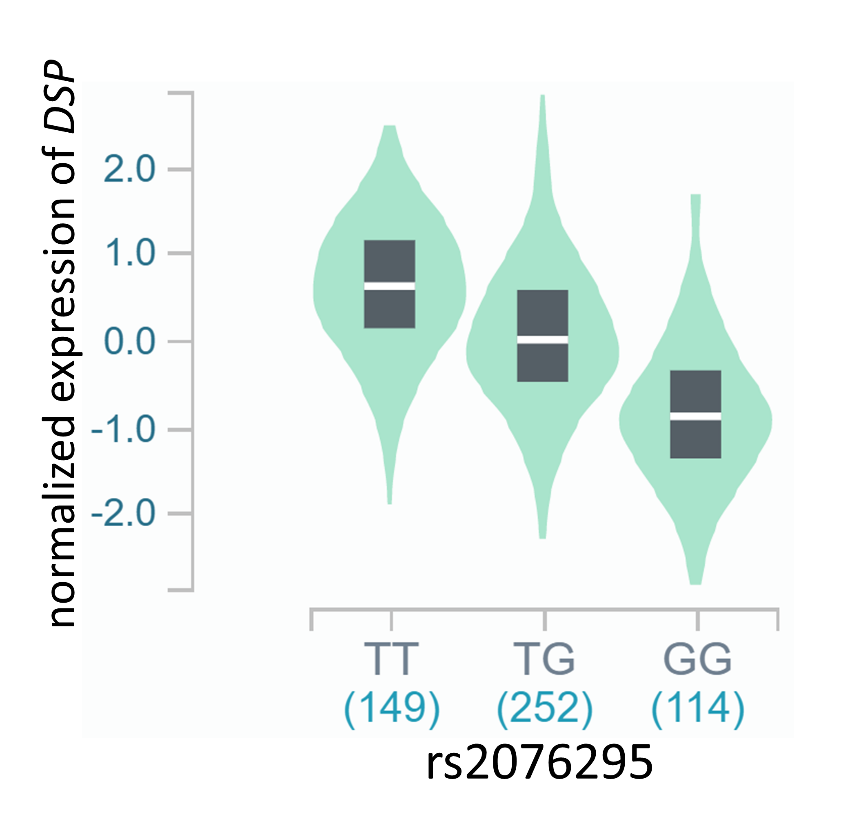


**Supplementary Figure S1**. *DSP* rs2076295G is associated with lower expression of *DSP* mRNA in lung.

eQTL data was obtained from the GTEx Portal database [36]. *DSP* rs2076295G was associated with decreased expression of *DSP* mRNA levels in lung (P= 3.7 x 10^-75^, normalized effect size = -0.73), where effect size is defined as the slope of the linear regression. Medians of normalized expression of *DSP* in rs2076295 T/T, T/G and G/G are 0.6030, -0.01700 and -0.8926, respectively.
